# *Bulletin* comment: In praise of the psychiatric ward round^†^

**DOI:** 10.1192/pb.bp.115.050799

**Published:** 2015-10

Facing a room full of strangers when hallucinating, delusional or suicidal could clearly be seen as an ordeal. However, such a situation continues regularly during ward rounds in psychiatric hospitals the world over. Campaigners advocating for the rights of patients or service users have repeatedly called for the trial of the weekly review to be abolished. It causes anxiety and stress, with most attendees complaining of feeling as though they had not been listened to and that vital information had been withheld from them.

Considerations of the power and more subtle dynamics between patients and staff – specifically the more senior clinicians – during the ward round provide helpful insights as to how damaging such regular interactions might be. The only conclusion surely must be the immediate cessation of such an anachronistic institutionalised process. However, the psychiatric ward must have some positive or beneficial aspects not only for a multitude of multidisciplinary members who attend it but also for the distressed, sick patient who has to endure it. Ward rounds have been taking place for decades; had they been purely detrimental they surely would have been junked years ago.

The chance for medical students and trainees doctors to see a senior clinician interview a patient in a difficult and challenging environment is invaluable. Observing such an interaction may offer junior staff insights into both beneficial methods that work and harmful approaches that should never be repeated. The opportunity to see signs and symptoms of psychiatric disease is educational for all.

Eliciting such phenomena may be harmful to the patient and the degree to which the assessment results in a negative experience is as much to do with the skill of the interviewer as it is with the patient's pathology.

Seeing how an individual deals with and reacts to a challenging situation often provides valuable insights as to any underlying psychopathology. The ward round might lead the patient with more personality-based difficulties to exhibit a pathognomonic response that might not have been otherwise observed. The previously elated patient with bipolar disorder who is able to sit in the ward round without breaking into song or rhyme is obviously improving.

And finally, following in the footsteps of Foulkes it might be argued that the social interactions of the patient in the group setting are providing some therapeutic benefit, however small.

